# Knowledge, Perceptions and Information about Hormone Therapy (HT) among Menopausal Women: A Systematic Review and Meta-Synthesis

**DOI:** 10.1371/journal.pone.0024661

**Published:** 2011-09-16

**Authors:** MinFang Tao, YinCheng Teng, HongFang Shao, Ping Wu, Edward J. Mills

**Affiliations:** 1 Department of Gynecology, Shanghai Jiaotong University, Affiliated Sixth People's Hospital, Shanghai, China; 2 Faculty of Health Sciences, University of Ottawa, Ottawa, Canada; 3 Department of Clinical Epidemiology & Biostatistics, McMaster University, Hamilton, Canada; Indiana University, United States of America

## Abstract

**Background:**

The use of hormone therapy (HT) by menopausal women has declined since the Women's Health Initiative randomized trial (WHI) in 2002 demonstrated important harms associated with long-term use. However, how this information has influenced women's knowledge and attitudes is uncertain. We aimed to evaluate the attitudes and perceptions towards HT use, as well as specific concerns and information sources on HT since the WHI trial.

**Method/Results:**

We did a systematic review to assess the attitudes and knowledge towards HT in women, and estimate the magnitude of the issue by pooling across the studies. Using meta-synthesis methods, we reviewed qualitative studies and surveys and performed content analysis on the study reports. We pooled quantitative studies using a random-effects meta-analysis. We analyzed 11 qualitative studies (n = 566) and 27 quantitative studies (n = 39251). Positive views on HT included climacteric symptom control, prevention of osteoporosis and a perceived improvement in quality of life. Negative factors reported included concerns about potential harmful effects, particularly cancer risks. Sources of information included health providers, media, and social contact. By applying a meta-synthesis approach we demonstrate that these findings are broadly applicable across large groups of patients.

**Conclusions:**

Although there are clear hazards associated with long-term HT use, many women view HT favorably for climacteric symptom relief. Media, as a source of information, is often valued as equivalent to health providers.

## Introduction

Hormone therapy has been one of the most broadly prescribed medications in recent memory. Although initially recommended for climacteric symptom control, there was a widespread understanding in the medical community that HT offered many favorable additional effects including cardiovascular and neurological protection [1], [2], [3]. because menopause naturally occurs when the ovaries begin decreasing production of estrogen and progesterone, and induced menopause occurs when the ovaries are surgically removed by bilateral oophorectomy or damaged by radiation or drugs, it usually takes 10 years for women to experience symptomatic changes. This is typically in a woman's late forties or early fifties, a time when lifestyle and other progressive diseases may become apparent. During this stage, many women experience physical and/or emotional symptoms [4], [5]. For some, symptoms related to menopause importantly impact their daily personal, professional, and social lives, resulting in a desire to reduce any adverse symptoms [6], [7].

With widespread support of HT for broad health benefits, single small trials or observational studies did not provide sufficient evidence to change the larger medical opinion. A large observational study, the Heart and Estrogen/progestin Replacement Study (HERS) [8] concluded that during 4.1 years of follow-up, treatment with oral conjugated equine estrogen plus medroxyprogesterone acetate did not reduce the overall rate of coronary heart disease (CHD) events in postmenopausal women with established coronary disease; on the contrary, it increased the rate of thromboembolic events and gallbladder disease. In 2002, the Women's Health Initiative (WHI) randomized trial involving over 26,000 women [9] was stopped early due to increased risks of invasive breast cancer, pulmonary embolism, CHD and stroke when compared with placebo, confirming the findings of the HERS study [10]. A meta-analyses of observational studies published in 2002 indicated that HT was associated with long-term important harms, and a benefit only at osteoporosis prevention and climacteric relief [11]. The follow-up WHI study demonstrated HT was responsible for breast cancer and excess deaths directly attributed to breast cancer [12].

Despite evidence from these large studies, as many as 50% of physicians remain skeptical toward the evidence from WHI and HERS, citing concerns with study designs and patient populations [13]. Further, industry involvement in clinician and patient education has led to concern that benefits of HT are being unnecessarily promoted and harms are being reduced [14] . For this reason, it may be challenging for health care providers and menopausal women to make informed decisions on HT use. Although the use of HT has decreased [15], [16], there is uncertainty among obstetrician-gynecologists that it may be a viable treatment option for climacteric symptoms relief such as hot flashes, vaginal atrophy, osteoporosis and loss of Libido [17]. While studies have examined the knowledge and perceptions of physician and healthcare providers about the evidence for HT [13], [17], [18], no systematic review about the overall attitude or perception of menopausal women towards HT exist. In order to summarize women's attitudes and knowledge regarding HT use, we applied a meta-synthesis of published studies [19], [20], [21], a strategy that permits reviewers to identify common perceptions and beliefs and to determine the magnitude of these beliefs with some confidence.

## Materials and Methods

### Study approach

Our study approach is based on a two-step analysis of the studies. Firstly, we will identify the themes that are identified in qualitative studies. Secondly, we will determine the magnitude of these themes in broader populations by conducting a meta-analysis of surveys that report the themes. We have published on these methods extensively in the past [19], [20], [21], [22], [23], [24].

### Eligibility criteria

For stage 1, we included two types of studies, qualitative studies and quantitative studies that used open-ended questions, allowing an unlimited number of participant responses. For stage 2, we included quantitative studies that report on the proportion of survey populations that affirm the issues raised in qualitative studies.

Eligible studies had to meet the following criteria: reported as an original research study and conducted in peri-menopausal women; contained information addressing attitude or knowledge towards HT; or information sources on HT. Studies that only compared the demographic characteristics between HT users and non-users; evaluated clinical outcomes; evaluated the proportion of HT use; or conducted within a specific population (eg. patients with breast cancer or cardiovascular diseases) were excluded.

### Search strategy

Using a formal search strategy, MFT and YCT searched the following databases independently and in duplicate (from 07/2002 to 01/2011): MEDLINE, AMED, Alt Health Watch, CINAHL, Nursing and Allied Health Collection: Basic, and the Cochrane Database of Systematic Reviews. Our search strategy used permutations and combinations of the following terms: *attitudes*, *perception*, *knowledge*, *behavior and belief*, *menopause*, *hormone treatment*, *HRT*, *qualitative*, *grounded theory*, *interview*, *questionnaire*, *cross-sectional* and, *survey*. In addition, we supplemented this search by reviewing the bibliographies of key papers. We worked together to assess relevant studies for inclusion and only English language studies were included. MFT and YCT independently reviewed the abstracts and chose the full articles after discussion.

### Data abstraction and validity assessment

MFT and HFS independently extracted data and appraised the validity. Disagreements were resolved by a third reviewer (PW). We extracted data on the methods of the studies using a modified checklist to assess internal validity [21] [25]. Quantitative studies were not scored as no accepted criteria exist for judging quality. A coding template to categorize key perceptions towards HT was developed iteratively during an initial review. This template consisted of the mutually exclusive headings listed as the positive factors that enable women to use; the negative factors that enable women to use; women' individual characteristics when making the decision and information sources of HT.

We then read all available surveys to determine whether they asked questions broadly representative of the themes identified in the qualitative studies. Data were regarded eligible for inclusion in the meta-analysis if the study reported proportions of respondents.

### Statistical analyses

We used the κ statistic to measure chance-adjusted agreement between reviewers for study eligibility. When information on proportions of respondents was available from the quantitative studies, we calculated weighted proportions of studies using the Freeman-Tukey method [26]. We calculated an overall estimate of effect by pooling the proportions of each quantitative study by applying a random-effects model, with 95% Confidence Intervals (CI) and lower CI truncated at zero. We assessed heterogeneity of proportions visually as pooling proportions always results in large estimates of heterogeneity and statistical techniques do not yet exist to interpret the extent of real between-study heterogeneity for proportions [27]. We used Stats Direct for all statistical procedures.

## Results

**Figure 1:**
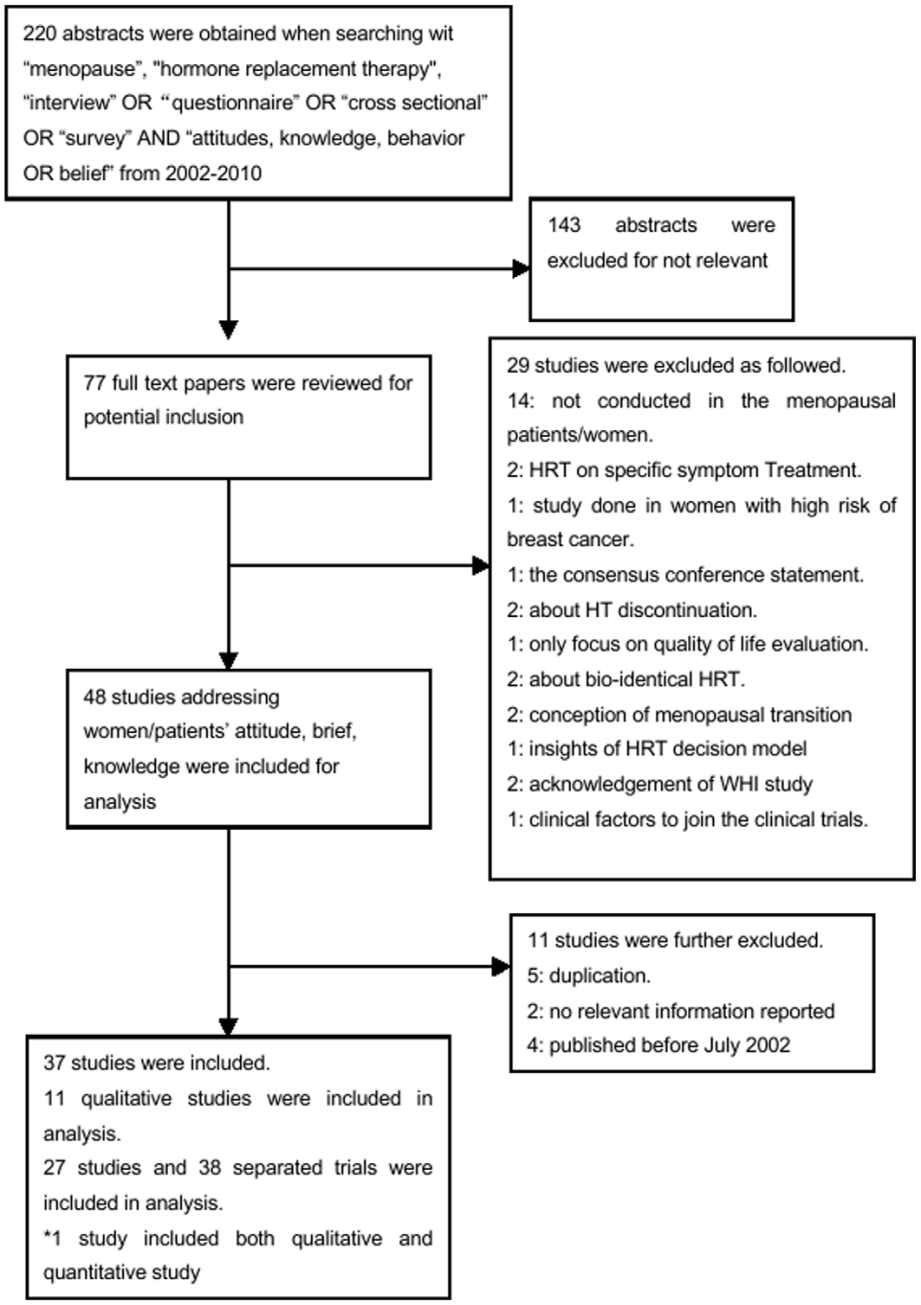
Flow diagram of included studies.

Our search identified 220 relevant abstracts. There was good (κ = 0.64) agreement between MFT and YCT on choosing the final 77 applicable full-text studies for potential inclusion. Of these studies, 40 were excluded for various reasons, leaving 37 studies [5], [7], [28], [29], [30], [31], [32], [33], [34], [35], [36], [37], [38], [39], [40], [41], [42], [43], [44], [45], [46], [47], [48], [49], [50], [51], [52], [53], [54], [55], [56], [57], [58], [59], [60], [61], [62] included in our analyses (See figure 1).

We included eleven qualitative studies [40], [53], [54], [55], [56], [57], [58], [59], [60], [61], [62] and 27 quantitative studies [7], [28], [29], [30], [31], [32], [33], [34], [35], [36], [37], [38], [39], [40], [41], [42], [43], [44], [45], [46], [47], [48], [49], [50], [51], [52], [63], reporting 31 independent studies. One study [40] included both qualitative and quantitative study. Table 1 shows the characteristics of the populations in the qualitative studies and Table 2 shows the methodology of these studies. Among these, 4 studies [40], [56], [60], [62] used focus groups (n = 248), five [53], [54], [57], [58], [59] used semi-structured interviews (n = 151), one [61] used both focus group and semi-structured interviews (n = 40) and one [55] used mainly open-ended questioning (n = 127). The attitudes identified towards HT are listed in Table 3.

**Table 1:** Study characteristics of qualitative study.

reference	country	population	Main focus of Paper	Setting	Main findings	Design
Walter2002^54^	UK	n = 40, (50–55 ys,)75% well- educated100% peri-menopause,32.5% never used HT.	Explore women's understanding of the risks associated with the menopause and HT.	general practices	Patients used their knowledge, risk perception and their individual belief system, experience, age and emotions to modify the salience of HT risk. Most of them favored communication with health providers. Sharing experience with the others would be important to facilitate in decision making	Focus group/semi-structured interviews
French2006^48^	USA	n = 127,(50–70 ys)100% well-educated,100% peri-menopause,14.2% never used HT	explore the impact of hormone therapy recommendations on patients' attitude and decision making	general practice office	HT should take into account women's preferences about symptom relief and the trade-offs among relevant risks. Emotional support during transitions in HT is encouraged	open ended
Ballard2002^46^	UK	n = 32, (51–57 ys)34.4% well-educated,100% peri-menopause,37.5% never used HT	explore women's perceived risk of menopause-related disease and the decision making of HT for disease prevention	community setting	Osteoporosis and heart disease are associated with decision to take HT, which are largely based on individual assessment of risk, but the value of HT is limited.	semi-structured interviews
Cifcili2009^47^	Turkey	n = 16, (42–53 ys)63% well-educated,100% peri-menopause,no data for HT use	explore women's knowledge of menopause and HT	gynecological clinic	Menopause is a natural transition process; seeking medical help is a way to cope with it. non-pharmacological options were favored because of HT side effects.	semi-structured interviews
Shelton2002^53^	USA	n = 75, (30–71 ys)100% well-educated,25.6% peri-menopause,37% never used HT	explore the attitude and belief about and pattern of HT use	community and clinic	Use of HT as either therapeutic or prevention is controversial. The target-oriented counseling, taking into account the individual attitudes toward HT, is expected	focus group
Loutfy 2006^33^	Egypt	n = 70, (50–59 ys)21.1% well-educated,100% peri-menopause,no one used HT	determine symptoms, perceptions and practices after natural menopause	community	Most participants had never heard about HT. Its cost and side-effects were a concern. Main information sources included the media.	focus group
Hepworth 2002^49^	Australia Adelaide	n = 21,(50–69 ys)(no data on education and HT use)100% peri-menopause,	explore the knowledge/attitude of HT and patients' willingness to participate in a long-term HT randomized control trial	general practices	HT was beneficial for symptom relief, “natural approach to health and anti-medication were expected, and more information about HT was expected.	focus group
Hyde 2010^50^	Ireland	n = 23, (42–63 ys)no data on education,100% peri-menopause,64.1% never used HT	explore women's experience of menopause and HT	Thematic Networks	HT effectiveness was in moderating bodily distresses.	semi-structured interviews
Kolip 2009^51^	Germany	n = 35, (46–75 ys)no data on education,100% peri-menopause,no one used HT	explore the reason why postmenopausal women undergo long-term hormone therapy	na	Target-oriented counseling is needed; the health providers should consider patients' individual attitudes toward menopause and HT.	semi-structured interviews
Weltom 2004^55^	England Scotland	n = 82, (50–69 ys)no data on education,100% peri-menopause,30% never used HT	explore the factors affecting HT decision making and the view about risk and benefits, attitude towards HT studyresults	general practice	Women regarded taking HT as highly personal; the reason for continuation was to improve quality of life regardless of the risks in the longer term.	focus group
Nekhlyudov 2009^52^	USA	n = 45, (45–60 ys)no data on education,100% peri-menopause,no one used HT	explore women's beliefs about hormone therapy and breast cancer risk	phone interview	To control menopausal symptoms was important and possibly outweighed the concerns about the potential risks of breast cancer.	structured interviews

**Table 2:** Reporting criteria of qualitative studies.

reference	Walter 2002 [61]	French 2006 [55]	Ballard 2002 [53]	Cifcili 2009 [54]	Shelton 2002 [60]	Loutfy 2006 [40]	Hepworth 2002 [56]	Hyde 2010 [57]	Kolip 2009 [58]	Weltom 2004 [62]	Nekhlyudov 2009 [59]
Was the data transcribed verbatim (ie. Were audiotapes, videotapes, used?	**✓**	**✓**	**✓**	**✓**	**✓**	**✓**	**✓**	**✓**	**✓**	**✓**	**✓**
If interview conducted, were questions predefined?	**✓**	**✓**	**✓**	**✓**	**✓**		**✓**	**✓**	**✓**	**✓**	**✓**
If focus group used, was the facilitator trained?	**✓**	**✓**			**✓**	**✓**	**✓**			**✓**	
Was saturation mentioned?		**✓**		**✓**			**✓**		**✓**	**✓**	
Was there a description of how the research themes were identified?	**✓**	**✓**	**✓**	**✓**	**✓**		**✓**	**✓**	**✓**	**✓**	**✓**
Were participants' answers reviewed for clarification?				**✓**			**✓**	**✓**	**✓**	**✓**	**✓**
Were sequences from the original data presented?	**✓**	**✓**		**✓**	**✓**			**✓**	**✓**	**✓**	**✓**
Were the findings analyzed by more than one assessor?	**✓**	**✓**	**✓**	**✓**	**✓**		**✓**	**✓**	**✓**	**✓**	**✓**

**✓** indicates the methodological item was mentioned in the original study.

**Table 3:** Attitude towards HT in qualitative studies.

reference	Walter 2002 [61]	French 2006 [55]	Ballard 2002 [53]	Cifcili 2009 [54]	Shelton 2002 [60]	Loutfy 2006 [40]	Hepworth 2002 [56]	Hyde 2010 [57]	Kolip 2009 [58]	Weltom 2004 [62]	Nekhlyudov 2009 [59]
**Positive factors that enable women to use**											
Effective for climacteric symptoms	**✓**	**✓**			**✓**		**✓**	**✓**	**✓**		**✓**
benefit outweighs risk		**✓**		**✓**						**✓**	
Osteoporosis prevention											
Treatment of menopause related disease			**✓**								
necessary supplement					**✓**				**✓**		
Improve quality of life					**✓**					**✓**	
MD recommendation		**✓**						**✓**	**✓**	**✓**	
**Negative factors that enable women to use**											
Potential side effects	**✓**	**✓**	**✓**	**✓**	**✓**	**✓**	**✓**	**✓**	**✓**	**✓**	
May cause cancer		**✓**	**✓**	**✓**	**✓**		**✓**	**✓**		**✓**	**✓**
May cause CHD											
Uncertain evidence	**✓**	**✓**		**✓**	**✓**				**✓**	**✓**	
No benefit or bad solution of HT		**✓**									
Distrust HT					**✓**						
Against person's natural healing process		**✓**			**✓**						
Experiment with my body					**✓**						
No knowledge about HT						**✓**					
Reduce life quality											
Not suggested by MD											
**Women's individual characteristics when making the decision**											
Preference for other treatment		**✓**		**✓**	**✓**		**✓**	**✓**		**✓**	**✓**
Unnecessary to use			**✓**							**✓**	
Personal experience, knowledge against HT use	**✓**	**✓**	**✓**							**✓**	**✓**
Dislike medication/HT isn't natural								**✓**			
Concern of the cost						**✓**					
Feel isolation when making decision		**✓**									**✓**
Medical history contraindicate HT use								**✓**			
Fear/Mistrust of research					**✓**					**✓**	
**Information sources**											
Media				**✓**	**✓**						
Work and social contact				**✓**	**✓**			**✓**			
Health professional				**✓**	**✓**			**✓**			
**Women's expectation**											
Communication with MD in decision making	**✓**				**✓**				**✓**	**✓**	
Belief that MD should make decisions	**✓**			**✓**				**✓**	**✓**	**✓**	
Favor evidence-based information	**✓**			**✓**			**✓**				
Balancing individualized situation	**✓**	**✓**	**✓**		**✓**				**✓**	**✓**	**✓**

**✓** indicates that the items was reported in the original text.

The characteristics of all quantitative studies are listed in Table 4 and whether they used structured questionnaires or structured interviews (n = 39,251) to determine the attitude and acknowledgement of HT (see Table 5 for the details). Studies were completed in Asia [7], [28], [29], [35], [48], [51], [63], Europe [7], [32], [34], [36], [38], [39], [43], [44], [45], [46], [49], North America [7], [30], [31], [37], [41], [42], [50], [52], South America [33], Latin America [7], Oceania [47] and the Middle East [40].

**Table 4:** Characteristics of quantitative studies.

reference	Num	country	age	Education(>9 ys)(%)	Response rate (%)	Never use HT(%)
Lam PM 2003 ^21^	978	Hong Kong	40–60	47	na	96
Kaur S 2004 ^22^	725	India	40–60	13	na	100
Barber CA 2004 ^23^	185	USA	25–84	72	98	100
Obermeyer CM 2004^24^	293	USA	45–55	98	62	71
Ekstrom H 2005 ^25^	1681	Sweden	45–60	49	76	59
Filho A 2005 ^26^	755	Brazil	> = 35	100	56	29
Hovi S 2005 ^27^	778	Finland	45–64	61	66	90
Chaopotong P 2005 ^28^	148	Thailand	>40	87	91	76
Thunell L 2005^1998 29^	4095	Sweden	> = 46	43	76	na
Thunell L2005^1992 29^	4504	Sweden	> = 46	77	76	27
Bosworth HB 2005 ^30^	533	USA	45–54	76	22	50
Genazzani AR 2006 ^31^	4201	Europe	45–60	79	na	58
Sveinsdottir H 2006 ^32^	561	Iceland	47–53	69	56	55
Loutfy I 2006 ^33^	450	Egypt	50–59	21	na	100
Twiss JJ 2007 ^34^	166	USA	40–55	99	na	54
Rigby AJ 2007 ^35^	781	USA	40–60	89	72	66
Uncu Y 2007 ^36^	1007	Turkey	39–89	13	na	83
Castelo-Branco C2007 ^37^	270	Spain	40–65	35	na	33
Lindh L 2007 ^999 38^	1180	Sweden	53–54	na	67	48
Lindh L 2007 ^2003 39^	1239	Sweden	53–54	68	72	56
Heinemann K 2008 ^3^	4791	Europe	40–70	19	70	62
Heinemann K 2008 ^3^	1500	USA	40–70	40	70	57
Heinemann K 2008 ^3^	3006	Latin America	40–70	9	na	80
Heinemann K 2008 ^3^	1000	Indonesia	40–70	4	na	98
Deeks A 2008 ^40^	692	Australia	45–55	na	77	na
Malik HS 2008 ^41^	102	Pakistan	40–75	“No education” 60.8	93	100
Donati S 2009 ^42^	720	Italy	45–64	44	74	84
Huston SA 2009 ^43^	689	USA	45–64	99	42	56
Jassim GA 2009 ^44^	260	Bahrain	30–64	86	na	97
Simon JA 2009 ^45^	961	USA	> = 35	69	na	59
Huang K 2010 [63]	1000	Asia	45–60	100	na	45

**Table 5:** Attitude towards HT identified in quantitative study.

reference	Deeks2008^40^	Twiss2007^34^	Heinemann2008^3^	Simon2009^45^	Jassim 2009^44^	Huston2009^43^	Lam2003^21^	kaur2004^22^	Thunell2005^29^	Malik2008^41^	Bosworth2005^30^	Chaopotong2005^28^	Lindh2007^39^	Huang 2010^56^	Deeks2008^40^	Twiss2007^34^	Heinemann2008^3^	Simon2009^45^	Jassim 2009^44^	Huston2009^43^	Lam2003^21^	kaur2004^22^	Thunell2005^29^	Malik2008^41^	Bosworth2005^30^	Chaopotong2005^28^	Lindh2007^39^	Huang 2010^56^
**Positive factors that enablewomen to use**																												
Effective for climacteric symptoms	**✓**		**✓**		**✓**		**✓**		**✓**			**✓**	**✓**	**✓**	**✓**		**✓**		**✓**		**✓**		**✓**			**✓**	**✓**	**✓**
benefit overweighs risk														**✓**														**✓**
Osteoporosis prevention	**✓**		**✓**		**✓**		**✓**		**✓**			**✓**	**✓**	**✓**	**✓**		**✓**		**✓**		**✓**		**✓**			**✓**	**✓**	**✓**
Treatment of diagnosed menopause related disease	**✓**		**✓**				**✓**						**✓**		**✓**		**✓**				**✓**						**✓**	
HT is a necessary supplement																												
Improve life quality							**✓**		**✓**			**✓**		**✓**							**✓**		**✓**			**✓**		**✓**
MD recommendation												**✓**														**✓**		
**Negative factors that enable women to use**																												
Potential side effects		**✓**	**✓**				**✓**		**✓**		**✓**	**✓**				**✓**	**✓**				**✓**		**✓**		**✓**	**✓**		
May cause cancer	**✓**		**✓**		**✓**		**✓**		**✓**			**✓**	**✓**	**✓**	**✓**		**✓**		**✓**		**✓**		**✓**			**✓**	**✓**	**✓**
May cause CHD	**✓**	**✓**			**✓**								**✓**	**✓**	**✓**	**✓**			**✓**								**✓**	**✓**
Uncertain evidence		**✓**	**✓**													**✓**	**✓**											
No benefit or bad solution of HT														**✓**														**✓**
No knowledge about HT		**✓**					**✓**			**✓**		**✓**				**✓**					**✓**			**✓**		**✓**		
Not suggested by MD		**✓**	**✓**						**✓**			**✓**				**✓**	**✓**						**✓**			**✓**		
Vaginal bleeding											**✓**														**✓**			
**Women's individual characteristics when making the decision**																												
Preference for other treatment		**✓**	**✓**	**✓**							**✓**					**✓**	**✓**	**✓**							**✓**			
Unnecessary to use			**✓**					**✓**	**✓**								**✓**					**✓**	**✓**					
Personal experience, knowledge against HT use																												
Dislike medication/HT is not natural		**✓**	**✓**													**✓**	**✓**											
Concerns of the cost			**✓**														**✓**											
Feels unsupported in decision making																												
Medical history contraindicate HT use			**✓**														**✓**											
**Information sources**																												
Media				**✓**		**✓**												**✓**		**✓**								
Work and social contact				**✓**		**✓**												**✓**		**✓**								
Health career				**✓**		**✓**								**✓**				**✓**		**✓**								**✓**

**✓** indicates that the items was reported in the original text.

To generalize the findings from qualitative studies, we pooled data for the factors reported in quantitative studies. From the pooled data we found that 47% (95%:34–60%) of participants perceived that HT was effective for climacteric symptoms, 26% (95% 15–40%) perceived that HT could be used in osteoporosis prevention and 33%(19%–48%) thought the benefits of HT outweigh the risks.

Thirty-one percent (95%CI: 18–46%) were aware of potential adverse events of HT, 37% (95%: 21–54%) aware HT may cause cancer and 14% (95%: 3–31% thought the risks of HT overweigh its benefit. Thirty-four percent (95%CI: 21–48%) thought it was unnecessary to use HT as the menopause-related symptoms were tolerable; 35% (95%CI: 24–47%) of respondents felt that the current evidence on HT was uncertain and 49%(95%CI: 22–76%) mentioned that they had no knowledge about HT.

**Figure 2:**
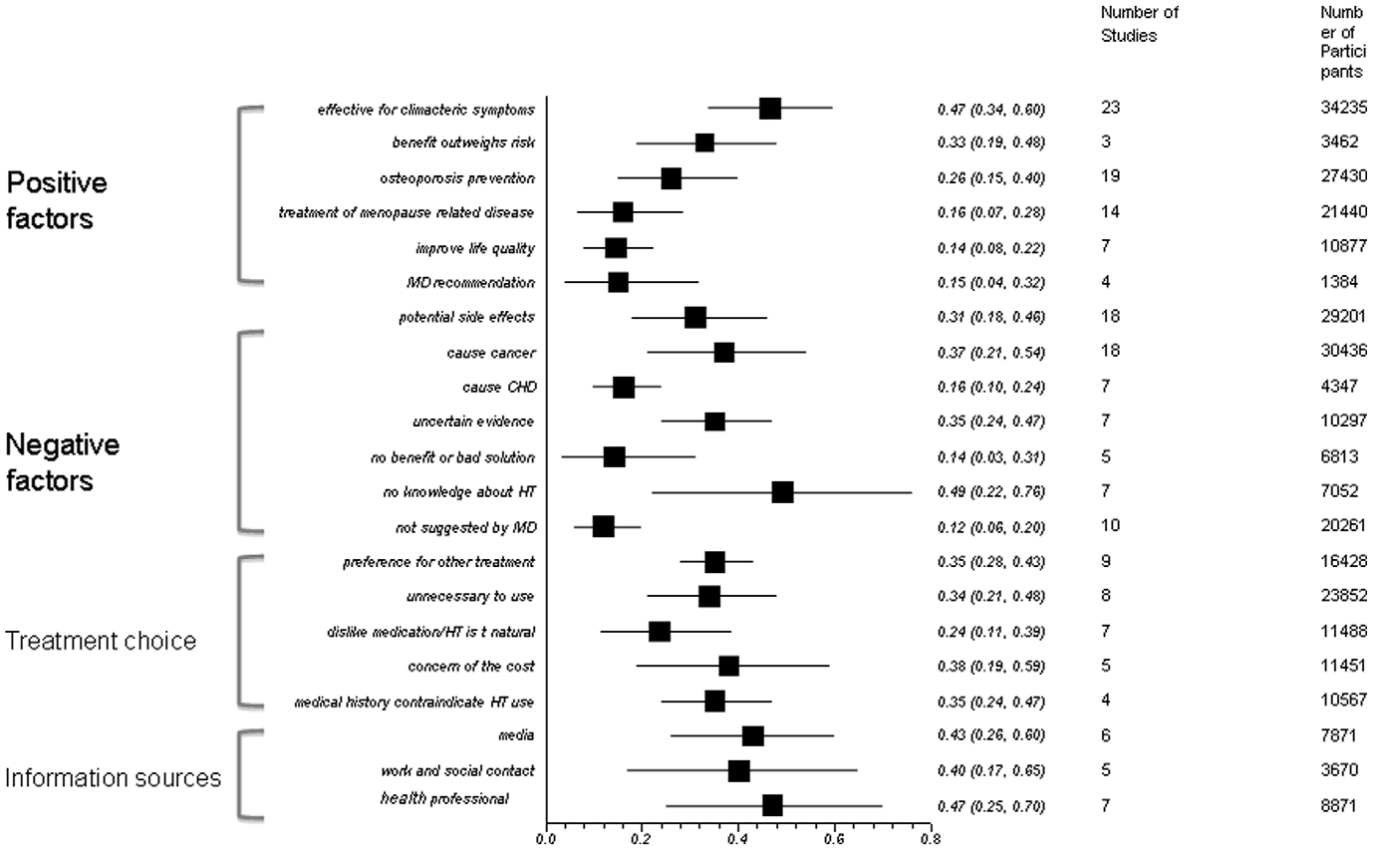
Pooled proportions of quantitative studies.

For information sources, forty-three percent (95%CI: 26–60%) obtained menopause- and HT related information from media including TV, internet, magazines, and newspapers, while 47% (95%CI: 25–70) obtained information from their health care provider and 40% (95%CI: 17–65%) from their work or social contacts. No knowledge of HT was evaluated in 7 studies, and 5 of them was conducted in developing countries,. We found visible heterogeneity in all pooled analyses, which we explain by our *a priori* hypotheses that findings from developed countries differ from developing countries in terms of knowledge. Figure 2 shows the pooled proportions of attitudes that were generated from the outcomes listed in Table 5.

## Discussion

In our current review, we summarized women's attitude and perceptions towards HT reported in studies published after the WHI and found somewhat low levels of concern about serious adverse events. In addition, with the development of information technology, we also found media sources share a similar status as healthcare and have become a commonly used form of information. Women in developing countries had lower levels of knowledge about HT than those in more developed settings. These findings are concerning for several reasons. First, many obstetrician-gynecologists have report they were unlikely to change their prescribing practice following the WHI [17]. And secondly, emerging evidence indicates sources of information on HT benefits and harms may be skewed according to the funding body supporting the messaging, potentially downplaying risks associated with HT [15].

There are both strengths and weaknesses to consider when interpreting our study. A novelty of our study is that we synthesized and conducted a meta-analysis based on issues raised in qualitative studies and then assessed the magnitude of these beliefs in larger populations by pooling the answers from the quantitative studies. We have used this approach several times previously on unrelated topics [19], [20], [21], [22], [23], [24] and have published its methodological assumptions [20], [22]. Limitations of our study lie most inherently in the reporting biases presented in the included qualitative studies. Unlike protocol driven studies, such as randomized trials, where the outcomes should be established prior to the conduct of the study, one cannot determine what issues will be conclusively raised in qualitative studies. Thus, it is possible that some issues raised by participants are not reported in the final manuscripts. For that reason, we believe our approach is specific but not necessarily sensitive, our approach to pooling utilized proportions, an infrequently used metric to apply meta-analysis to. Although several methods of weighting proportions exist, we used the Freeman-Tukey method as we have evaluated its performance previously in meta-analysis [23], [64]. The choice of weighting proportions approach does not change the results of a meta-analysis importantly. We assessed heterogeneity visually and explained heterogeneity using a priori explanations of heterogeneity, specifically, geographic location of the study. Common methods of assessing heterogeneity do not perform well with proportions and appear to overestimate heterogeneity even when it is low [27].

Although in 2011, the use widespread use of HT seems misguided, HT has been broadly applied in clinical medicine for several decades. There is, however, consensus that HT is effective at climacteric symptoms reduction and osteoporosis prevention [65], [66]. Although many women benefit from intermittent HT use, the concerns about longer-term adverse events frequently outweigh the short term benefits. In 2005, the boards of the international, the Asian pacific, the European and the North American menopause society conducted post-hoc analyses of the WHI trial and noted that advanced age of the participants was importantly associated with adverse events. Concern about adverse events has also diminished the use of HT. HT use has declined by up to 62% since the WHI [67], [68]. In addition to decreased HT use, several locations have witnessed a potentially associated decrease in breast cancers.

It is important to recognize that it is difficult for patients to make informed decisions as many people obtain their health information from the media, particularly the internet. The issue is that websites can be of variable quality and may promote anti-evidence-based information [69] or may diminish the risks of HT while highlighting the benefits, or vice versa. As healthcare providers, physicians should either initiate or engage in discussions raised by the patients about HT, and recognize that this is an important opportunity to guide patients to evidence-informed sources of information, such as National women's health Resource Center, a non-profit resource aimed at providing up-to-date information for women. For women requesting HT, physicians may consider informing women that climacteric symptoms may be short-lasting and benign, and should be aware of the balanced effectiveness and risks of HT based on their individual situations [4].

In conclusion, HT has important adverse events, especially towards breast, many menopausal women are uncertain about the benefits and risks of HT indeed. Sources of reliable information may be an important challenge for patients. As health care providers, it is important to discuss both the benefits and risks with women and make the decision based on their culture, personal experience and readiness, especially in the developing countries. Providing a standard protocol for administration of HT, routine follow-up health examinations and instituting health teaching prior to prescription may be reasonable steps to assure HT is appropriately used and women remain informed and protected.
